# Early effects of a novel 5-HT_4_ agonist (PF-04995274) and the SSRI citalopram on emotional cognition in unmedicated depression: the RESTAND study

**DOI:** 10.1192/bjp.2026.10664

**Published:** 2026-05-28

**Authors:** A.L. Gillespie, A.N. de Cates, J. Scaife, M. Blandhol, M.A.G. Martens, D Gibson, B.R Godlewska, W Howard, P.J. Cowen, S.E. Murphy, C.J. Harmer

**Affiliations:** 1 University Department of Psychiatry, Warneford Hospital, University of Oxford, Oxford, UK; 2 Oxford Health NHS Foundation Trust, Warneford Hospital, Oxford, UK; 3 Institute for Mental Health, University of Birmingham, Edgbaston, Birmingham, UK; 4 Department of Physics and Computational Radiology, Oslo University Hospital, Norway; 5 Faculty of Medicine, University of Oslo

## Abstract

**Background:**

Selective serotonin reuptake inhibitors (SSRIs) are limited by inadequate response in a significant proportion of patients, slow onset, minimal cognitive benefit, and side effects. Preclinical studies suggest selective serotonin 4 receptor (5-HT_4_R) agonists may produce faster antidepressant effects via distinct mechanisms, however there has been no experimental research in clinical populations to date.

**Aims:**

To test whether the novel 5-HT_4_R partial agonist PF-04995274 produces early behavioural and neural changes in emotional cognition similar to SSRIs in patients with unmedicated major depressive disorder (MDD).

**Method:**

In a double-blind, placebo-controlled trial, 90 participants with MDD were randomised to 7 days of PF-04995274 (15 mg), citalopram (20 mg), or placebo. Emotional processing was assessed using a behavioural facial expression recognition task and fMRI of implicit emotional face processing (days 6–9). Observer- and self-reported symptoms of depression were also measured at baseline and study end.

**Results:**

As anticipated, citalopram reduced relative accuracy and increased relative reaction time to identify negative faces, with corresponding changes in neural activity (reduced left amygdala activation to emotional faces and valence-specific shifts in cortical regions). In contrast, PF-04995274 produced no change in behavioural negative bias or amygdala activity but increased medial-frontal cortex activation across valences. While this was not a clinical trial, both active treatments demonstrated an early treatment response with reduced observer-rated depression severity relative to placebo; PF-04995274 also reduced self-reported depression, state anxiety, and negative affect.

**Conclusions:**

PF-04995274 did not show the typical antidepressant profile of negative bias reductions observed with citalopram. Instead, it was associated with distinct increased medial-frontal activation during an emotional faces task, coupled with preliminary evidence of early clinical improvement, suggesting a potential alternative pathway for antidepressant effects. Findings support further clinical trials of 5-HT_4_R agonists and investigation of pro-cognitive and mood effects.

**Clinicaltrials.gov registration number:**

NCT03516604.

**Data set information:**

Analysis scripts and anonymised behavioural data are available on OSF.

## Introduction

### The need for novel antidepressants

Selective serotonin reuptake inhibitors (SSRIs) are the most commonly prescribed antidepressants for major depressive disorder (MDD). First line treatment with SSRIs effectively reduces depressive symptoms in approximately half of patients with depression, but many others experience an insufficient response(1). SSRIs are also often limited by a slow onset of therapeutic effect(2, though see 3,4 for evidence of early treatment effects), common side effects such as sexual dysfunction(5, though see 6 for improvements in some patients), and limited benefit for cognitive impairment(7). Considering the global prevalence of MDD(8), identifying alternative treatments to tackle these limitations is essential.

### Emotional processing model of antidepressant action

A key mechanism of SSRI action is their ability to ameliorate negative biases in information processing common to MDD(9), such as greater recall of negative (vs positive) information and a greater likelihood of interpreting ambiguous stimuli negatively(10). The SSRI citalopram, for example, reduces negative biases in the recall of self-referential words and the interpretation of facial expressions(11), and attenuates neural response to negative faces in the amygdala and associated networks(12). These changes occur early in treatment, are associated with later symptom improvement, and are seen across several existing antidepressant treatments with different pharmacology, suggesting they may reflect a common or converging mechanism (13,14). Emotional processing models have therefore been used in antidepressant drug development as an early biomarker of antidepressant action(15).

### The potential of serotonin 4 receptor (5-HT_4_R) agonists

One approach for developing novel antidepressant treatments is selective targeting of serotonin receptor subtypes involved in mood and cognition. Of particular interest is the potential of selective serotonin 4 receptor (5-HT_4_R) agonists in the treatment of depression and cognition(16). Positron emission tomography (PET) studies have shown reduced 5-HT_4_R binding in MDD(17) and preclinical work demonstrates that 5-HT_4_R agonists produce antidepressant- and anxiolytic-like effects in rodent models, including the forced-swim test, sucrose preference, and elevated plus maze (18–20). Notably, these studies found effects consistently occur more rapidly with 5-HT_4_R agonism than with SSRIs (2-3 times faster), which may be due to a rapid induction of neuroplastic changes, and the direct excitation of serotonin cells in the dorsal raphe nucleus (e.g. 21). Together, these findings suggests that the 5-HT_4_R is a promising fast-acting antidepressant target.

### Aim of current study

The current experimental medicine study aimed to investigate the translation of these preclinical antidepressant effects of 5-HT_4_R agonism to human models of antidepressant action, in participants with un-medicated major depressive disorder. Specifically, we used a novel, highly selective 5-HT_4_R partial agonist (PF-04995274) to investigate early effects (6-9 days) on behavioural and neuroimaging models of emotional cognition previously shown to be sensitive to the effects of SSRIs. We hypothesised that PF-04995274 would reduce negative emotional biases and reduce the neural response to negative stimuli, similar to the effects seen with SSRIs. We included a group randomised to citalopram as a positive control to confirm expected SSRI-induced changes. As this was the first investigation of 5-HT_4_R agonism in MDD, we also measured effects on depression symptomatology.

## Methods

### Participants

Between 1^st^ August 2018 and 29^th^ July 2022, ninety participants (aged 18-61) with Major Depressive Disorder (DSM-5) were recruited. Diagnosis was confirmed by study psychiatrists, using the Structured Clinical Interview for DSM Disorders (SCID). Participants had received no drug or face-to-face psychological treatment for their depression within the previous six weeks, and were not taking any other psychotropic medication. Participants were adults of any gender, fluent in English, with no contraindications to 5-HT_4_R agonism or citalopram. We excluded potential participants who had failed to respond to antidepressant medication in their current episode of depression, or were at clinically significant risk of suicide. We also excluded those who may be at increased risk of adverse events (e.g., severe gastro-intestinal problems). See Supplementary Materials for a full list of inclusion and exclusion criteria.

The authors assert that all procedures contributing to this work comply with the ethical standards of the relevant national and institutional committees on human experimentation and with the Helsinki Declaration of 1975, as revised in 2013. All procedures involving human subjects/patients were approved by the South-Central NHS Research Ethics Committee (18/SC/0076). The protocol was pre-registered with clinicaltrials.gov (NCT03516604). Participants gave written and verbal informed consent. Participants were paid £200 for their participation (or £170 if the MRI scan was omitted due to contraindication).

### Study design

This was a between-subject, double-blind, randomised, placebo-controlled experiment. Participants received 7 days of PF-04995274 (15 mg once daily), citalopram (20 mg once daily), or placebo, taken orally; up to two additional days’ dosing was allowed to accommodate scheduling.

The study included four visits in total: (a) Screening; (b) Dosing Visit; (c) Research Visit One (including fMRI scan) on day 6-9, and (d) Research Visit Two (including all cognitive tasks and end of study symptom assessment) on day 7-9 (Figure S1). By day 6 when scanning took place, steady state plasma levels were expected; the half-life (t_1/2_) of PF-04995274 is ~30 hours, and citalopram is ~35 hours. All visits occurred at the Warneford Hospital, Oxford University Department of Psychiatry. Scanning took place at the Oxford Centre for Human Brain Activity (OHBA), part of the Oxford Centre for Integrative Neuroimaging (OxCIN) (previously Wellcome Centre for Integrative Neuroimaging, WIN).

### Interventions

PF-04995274 is a highly-selective 5-HT_4_R partial agonist, developed by Pfizer, and provided (alongside matched placebo tablets) via the MRC-Industry Asset Sharing Initiative. It has been previously tested in human clinical trials of healthy volunteers, with PET studies establishing that 5mg of PF-04995274 produced > 80% receptor occupancy of brain 5-HT_4_R four hours after the first dose, and that 15mg was well-tolerated (22).

Citalopram is a selective-serotonin reuptake inhibitor (SSRI), and is a safe, well-tolerated, licensed treatment for MDD. It was provided and encapsulated (alongside matched placebo capsules) by Cardiff and Vale University Health Board, St. Mary's Pharmaceutical Unit (SMPU).

The first dose was administered on site, with participants monitored for 3 hours post-dosing. All subsequent administration occurred at home.

### Randomisation and blinding

The research team and participants were blinded to group allocation. The randomisation code (stratified by gender) was drawn up and held by Oxford Health Foundation Trust Pharmacy (Clinical Pharmacy Support Unit – CPSU – Kennington), using an online randomisation tool (Sealed Envelope); they also stored and dispensed the medication. Group allocation was concealed using sequential numbered containers (see Supplementary Materials for more details).

### Power calculation

Based on previous citalopram–placebo comparisons (23), 19 participants per group would provide 90% power (α = 0.05) for behavioural measures. To accommodate potential exclusions, subtler effects in fMRI analyses, and the less well-characterised effects of 5-HT_4_R agonism, we aimed for 25 participants with complete behavioural and fMRI data per group. Including 13 participants who completed behavioural assessments but not MRI (due to contraindications), 90 participants were recruited.

### Behavioural measure of emotional cognition

Emotional cognition was assessed at Research Visit Two (day 7-9) with a battery of cognitive tasks from the Emotional Test Battery (P1vital® Limited Products)(24), including the Facial Expression Recognition Task (FERT): Participants viewed briefly presented (500 ms) faces depicting basic emotions (happiness, fear, anger, disgust, sadness, surprise, neutral) at varying intensities and were asked to classify each via button press. There were 250 trials in a fixed pseudorandomized order.

(See Supplementary Materials for details of other emotional cognition tasks. Results from non-emotional cognition tasks are reported elsewhere (25)).

### Neuroimaging measure of emotional cognition

Neural activity during emotional processing was assessed using an implicit emotional cognition task conducted during an fMRI scan. Briefly, emotionally-valenced faces (fearful or happy) were presented on screen for 100ms. Participants were instructed to indicate the assumed gender of the face (male or female) as quickly as possible using a button press. No reference was made to face emotion during the instructions. There was a rest block at the start of the experimental run, and then the task followed an A-B-Rest design (condition A, fearful faces, 18s; condition B, happy faces, 18s; rest, 12s). Seven repetitions yielded 126s per emotional condition and 96s rest. This version was a recent modification of the task, which we have shown to be sensitive to the acute effects of antidepressants on neural processing(26). The task software was written using Psychopy version 1.84.2.

Blood-oxygenation-level-dependent (BOLD) fMRI and T1-weighted anatomical images were acquired using a 3-Tesla Siemens Prisma scanner, equipped with a 32-channel head matrix coil (Siemens, Erlangen, Germany). Foam padding and a head restraint were used to control head movement. In the scanner, participants also completed a memory encoding task and resting-state scan (reported elsewhere (25)), and an arterial spin-labelling (ASL) scan. The full acquisition protocol, along with radiography procedure, is available from the Open WIN MR Protocols database here: 10.5281/zenodo.6107724.

### Clinical assessment and questionnaire measures

To obtain an observer-related measure of depression severity, trained researchers assessed participants at baseline and end-of-study using the 17-item Hamilton Rating Scale for Depression (HAM-D)(27).

To obtain a self-report measure of depression severity, participants completed the Beck Depression Inventory-II(28) at baseline and end-of study, as well as the Snaith-Hamilton Pleasure Scale (SHAPS)(29) to measure anhedonic symptoms. At baseline, the Spielberger State-Trait Anxiety Inventory, Trait Version (STAI-T)(30) and Eysenck Personality Questionnaire(31) were also completed, to assess baseline trait anxiety and personality. State affect and anxiety were measured at both research visits using: the Positive and Negative Affect Scale (PANAS)(32); visual analogue scales (VAS)(33); and the Spielberger State-Trait Anxiety Inventory, State Version (STAI-S)(30). Commonly reported side effects (checklist in Supplementary Materials) were also measured at each visit, and at home across the study period. At the end of the study, participants were asked to guess their group allocation with a multiple-choice question.

### Statistical analysis

#### Behavioural and clinical data

R (version 4.3.3) was used for all data processing and analysis. Scripts and data are publicly available.

For all behavioural data, outliers were determined based on blinded visual inspection of histogram plots. Outlier removal/sensitivity checks were determined on 12^th^ January 2023, prior to unblinding. There was determined to be one outlier participant in the FERT data, who was removed during sensitivity checks.

Demographic characteristics and baseline clinical measures are reported descriptively. For all analyses, the citalopram group was first compared to the placebo group to confirm expected SSRI effects. The 5-HT_4_R group was then compared to placebo to determine the effect of PF-04995274. Group differences in cognitive task performance and clinical outcomes were assessed using ANOVA, with group as between-participant factor and task condition (e.g. valence) as within-participant factor. For scales where baseline measures were collected (HAM-D, BDI and SHAPS), baseline severity was included as a covariate. Chi-square tests were used to assess group differences in number of reported side-effects. A p value less than 0.05 was used to denote statistical significance. Partial eta-squared is reported as a measure of effect size.

As the STAI-S, PANAS and VAS measures were not completed an equal number of times for all participants (due to differences in MR scanning compatibility and thus completion of one or two research visits), an average score across research visits was calculated for each participant to indicate overall end-of-study affect.

#### Magnetic Resonance Imaging Data

fMRI data were pre-processed and analysed using FEAT (FMRI Expert Analysis Tool), version 6.0.4, part of FSL (FMRIB’s Software Library2). See Supplementary Material for pre-processing steps.

In the first-level analysis, individual activation maps were computed using the general linear model with local autocorrelation correction. Three explanatory variables were modelled: “happy” and “fear” images and explicit fixation cross, to replicate the models in our previous papers(26). Temporal derivatives were included in the model. Variables were modelled by convolving each block with a haemodynamic response function with a standard deviation of 3s and a mean lag of 6s. Two participants had significant movement (one allocated to citalopram, one allocated to placebo) and were therefore excluded from analysis prior to unblinding. All other absolute displacements were less than 2.2 voxels and relative displacements less than 0.2 voxels. FSL motion outliers tool was used to reduce the influence of remaining motion. At the whole-brain level, we contrasted fearful images with happy and fixation cross: (1) fear > fixation; (2) happy > fixation; (3) fear > happy; (4) happy > fear; (5) mean (of happy and fearful faces) > fixation.

In the second-level analysis, whole-brain individual data were combined at a group level (placebo vs. active drug - either citalopram or PF-04995724) using a mixed-effects group analysis across the whole brain corrected for multiple comparisons. Groups were contrasted with each other using the following comparisons: (1) placebo > active; (2) active > placebo; (3) mean of active + placebo participants. Brain activations showing significant group differences were identified using cluster-based thresholding (Z > 3.1, p < 0.05 FWE corrected). Significant interactions from whole-brain analyses were further explored by extracting percentage BOLD signal change with Featquery for each type of contrast.

As the amygdala(34) and prefrontal cortex(35) were a particular focus of our hypothesis, the amygdala, medial frontal cortex (MFC), and orbitofrontal cortex (OFC) were pre-specified as regions of interest (ROI). A functional ROI mask was created for each by multiplying mean activation (mean>fixation contrast) for all participants (placebo, citalopram and PF-04995274) by the Harvard-Oxford subcortical atlas anatomical mask at a 50% threshold. Percentage BOLD signal change for each contrast in each hemisphere was then extracted.

## Results

### Sample demographics

90 participants completed the full period of study medication and the final research visit. 77 of these had no scanning contraindications and completed an MRI scan visit prior to their final research visit. Participants were predominantly white, native English speakers aged 20-40, with education equivalent to completion of an undergraduate degree (Table 1, and Table S1 for comorbidities).

### On a behavioural measure of emotional cognition, participants who received the 5-HT_4_ agonist did not show the reduced negative bias seen with citalopram

**Figure 1:**
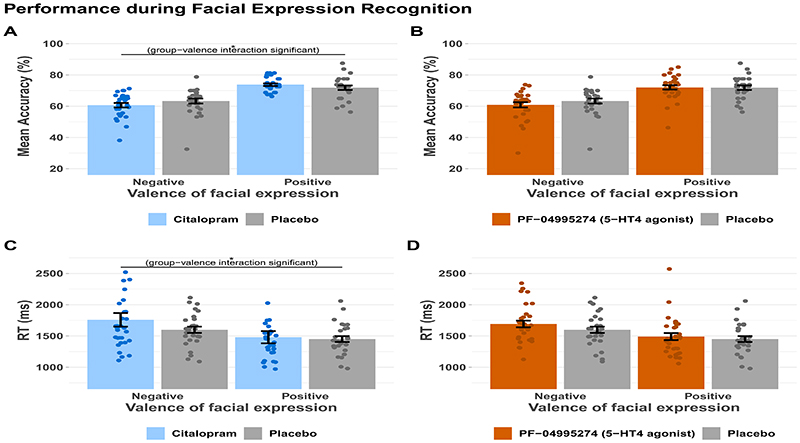
**In a Facial Expression Recognition Task, only the citalopram vs placebo group comparison showed a significant interaction with valence on accuracy and reaction; compared to placebo, the citalopram group displayed reduced accuracy and slower reaction times to identify negative faces relative to positive faces. A** - citalopram vs placebo, comparing percentage accuracy for negative vs positively valenced faces. **B** – PF-04995274 vs placebo, comparing percentage accuracy for negative vs positively valenced faces. **C** - citalopram vs placebo, comparing reaction time for negative vs positively valenced faces. **D** – PF-04995274 vs placebo, comparing reaction time for negative vs positively valenced faces * = p < 0.05, for interaction between group and valence on ANOVA. Errors bars show standard error of the mean. Grey = placebo, Blue = citalopram, Orange = PF-04995274. Placebo group data is shown in both citalopram vs placebo plots (A and C), and in PF-04995274 vs placebo plots (B and D).

When comparing citalopram to placebo, there was no main effect of group (F(1,56)=0.49, p=0.49, ηp2=0.009) but there was a significant interaction between medication group and valence on total percentage accuracy of emotional face classification (F(1,56)=4.30, p=0.043, ηp2 =0.07). Consistent with previous reports, the citalopram group showed relatively greater accuracy for positive faces and reduced accuracy for negative faces, compared to placebo (Figure 1-A).

Similarly, when comparing reaction times for the citalopram group to placebo, there was no main effect of group (F(1,56)=0.98, p=0.325, ηp2=0.02) but there was a significant interaction between group and valence on speed of emotional face classification (F(1,56)=6.78, p=0.012, ηp2=0.11), with the citalopram group showing slower reaction times for negative faces (Figure 1-C).

In contrast, when comparing the PF-04995274 group to placebo, there was no main effect of group on accuracy (F(1,57)=0.76, p=0.39, ηp2=0.01) or reaction time (F(1,57)=1.14, p=0.289, ηp2=0.02), and no interaction between group and valence for accuracy (F(1,57)=1.09, p=0.301, ηp2=0.02) or reaction time (F(1,57)=1.39, p=0.244, ηp2=0.02) (Figure 1-B/D).

The significance, size and pattern of all results remained consistent in all sensitivity checks (using an adjusted accuracy measure which accounts for response bias, transforming the data to address skew, removing a potential outlier participant). See Supplementary Materials for results on misclassifications rates.

### On a neuroimaging measure of emotional cognition, participants who received the 5-HT_4_ agonist showed a distinct pattern of neural changes to those on citalopram, with increased medial-frontal activation across emotional face valences

#### Whole brain analyses

When comparing citalopram to placebo, there was no difference for mean of emotional faces (fear+happy) vs fixation, but there was significant differential activation for fear vs happy in six clusters covering angular gyrus, lateral occipital cortex, cerebellum, middle temporal gyrus and fusiform gyrus. In all clusters, the placebo group showed greater activation for fearful faces and the citalopram group showed greater activation for happy faces (p<0.01, Figure S2 and Table S6). When comparing the PF-04995274 group to placebo, there were no significant differences on any contrast.

For behavioural data (accuracy and reaction time for gender discrimination) and broad task effects in the fMRI data, see Supplementary Materials.

#### ROI analyses

**Figure 2:**
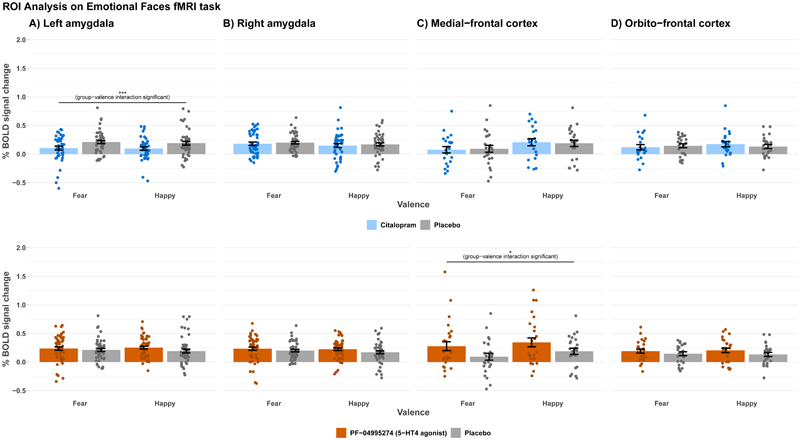
**In ROI analysis of an implicit emotional faces fMRI task, citalopram was associated with reduced amygdala BOLD response across valences and PF-04995274 was associated with increased medial-frontal BOLD response across valences. Figure shows % mean BOLD signal change in A) left amygdala, B) right amygdala, C) medial frontal cortex, and D) orbito-frontal cortex, defined using functional mask**. Values extracted separately for fearful > fixation and happy > fixation, comparing citalopram group vs placebo group and then PF-04995274 group vs placebo group. *** = p < 0.001, for interaction between group and valence on ANOVA. Errors bars show standard error of the mean. Grey = placebo, Blue = citalopram, Orange = PF-04995274. Placebo group data is shown in both citalopram vs placebo plots (upper row), and in PF-04995274 vs placebo plots (lower row).

In the left amygdala, when comparing citalopram to placebo, there was a main effect of group (F(1,90)=4.008, p=0.048, ηp2=0.04), but no significant interaction between group and face valence on % BOLD signal change (F(1,90)=0.001 p=0.97, ηp2=0.0001) i.e. the citalopram group showed reduced amygdala BOLD response across valences (Figure 2 - A). There was no main effect or interaction for the right amygdala (p>0.4). When comparing the PF-04995274 group to placebo, there was no significant main effect or interaction (p>0.15) for the left nor right amygdala (p>0.08).

In the medial-frontal cortex, there was a main effect of group when comparing the PF-04995274 group to placebo (F(1,100)=5.98, p=0.016, ηp2=0.06), but no significant interaction between group and face valence (F(1,100)=0.042, p=0.84, ηp2=0.0004) i.e. the PF-04995274 group showed greater BOLD response across valences (Figure 2 - BError! Reference source not found.). There was no main effect or interaction when comparing citalopram group to placebo (p>0.7).

In the orbito-frontal cortex, there was no significant difference for any group comparison, on any contrast (Figure 2 - C).

### After one week, participants given either PF-04995274 or citalopram showed significant decreases in depressive symptoms

#### Observer-rated symptoms

At baseline, the mean HAM-D score for the whole sample was 16.1 (sd=2.78), indicating mild to moderate depression(36). Groups were reasonably well matched, with marginally lower severity in the citalopram group (m=15.2, sd=2.8) compared with the placebo (m=16.9, sd=2.88) and PF-04995274 (m=16.2, sd=2.51) groups.

Controlling for baseline severity, the citalopram group scored significantly lower on the HAM-D at the final research visit (m=12.7, sd=5.20) compared to placebo (m=15.5, sd=3.9) (F(1, 54)=5.50, p=0.02, ηp2 = 0.09), as did the PF-04995274 group (m=12.7, sd=4.04) (F(1, 56)=7.61, p=0.008, ηp2 = 0.12) (**Error! Reference source not found.Error! Reference source not found**.). The significance and size of these effects remained, or increased, in sensitivity analyses (removing the sleep sub-scale, or removing potential outlier participants), both groups scored significantly lower if analysis was restricted to core depressive sub-scale, and only the PF-04995274 group scored significantly lower on the anxiety sub-scale (Table S2).

#### Self-reported symptoms and affect

Controlling for baseline severity, the PF-04995274 group - but not the citalopram group – reported significantly lower depressive symptoms (BDI) at the final visit than placebo. Similarly, only the PF-04995274 group reported lower state anxiety (STAI-S) than placebo at the final visit. Both groups reported significantly lower negative affect at final visits compared to placebo, with double the effect size in the PF-04995274 vs citalopram group. Only the citalopram group reported significantly lower positive affect at final visits compared to placebo. Neither group significantly differed from placebo on any other scales. See Supplementary Materials – Table S3 and S4 for all statistics.

#### Success of blinding

There was a significant association between medication group and guessed medication (*X*^*2*^ (6, N=90)=12.92, p=0.04), driven by the citalopram group (citalopram vs placebo, *X*^*2*^ (3, N=59)=8.09, p=0.04; PF-04995274 vs placebo, *X*^*2*^ (3, N=60)=2.06, p=0.56). 43.34% of participants allocated to citalopram correctly guessed their allocation (vs 9.78% of those allocated to PF-04995274), and 60% correctly guessed they had an active drug (vs 25.81% of PF-04995274 participants).

#### Side effects

PF-04995274 group reported new fatigue significantly more often (X^2^=6.5, p=0.04), all reports of which were described as mild-moderate. There were no other significant group differences in new reported side effects (p>0.1), though only participants in the PF-04995274 group reported increased appetite (not systematically recorded) (see Supplementary Materials – Table S5).

Five participants (two on placebo, two on citalopram, one on PF-04995274) withdrew from the study due to adverse events, all of which resolved within 24 hours of discontinuation.

## Discussion

The primary finding of this double-blind, randomised study is that unmedicated depressed participants who received the novel 5-HT_4_R agonist (PF-04995274) did not display the profile of behavioural and neural changes in emotional cognition observed with citalopram, and anticipated based on previous SSRI research(11,12). However, participants who received the 5-HT_4_R agonist did show distinct neural changes during emotional cognition, and both groups reported significant reductions in depressive symptoms after one week of administration compared to placebo.

On a behavioural facial expression recognition task, participants randomised to citalopram displayed indications of ameliorated negative bias; they showed relatively reduced recognition of - and slower reaction times to - negative faces, and increased recognition of - and faster reaction times to - positive faces, compared to the placebo group. Concurrently, in an implicit emotional faces fMRI task, whole brain analyses revealed significantly reduced activity in response to fearful vs happy faces, compared to placebo, across a number of relevant brain regions including angular gyrus, lateral occipital cortex, middle temporal gyrus, and fusiform gyrus. Additionally, pre-registered ROI analysis showed reduced left amygdala activation to emotionally-valenced faces in the citalopram group. Collectively, these findings align with previous literature on neural effects of SSRIs during emotional processing, which are thought to be a key mechanism of SSRI efficacy(9).

In contrast, the 5-HT_4_R agonist group showed no significant differences on any behavioural measures of emotional cognition or in amygdala BOLD response to emotional stimuli, and no valence-specific neural changes. This broadly aligns with our previous research using 1 and 6 days of the 5-HT_4_R agonist prucalopride (1mg). However, in ROI analysis, the 5-HT_4_R agonist group did demonstrate significantly increased medial-frontal cortex activation in response to both negative and positive faces. The medial-frontal cortex modulates amygdala activity, and is thought to be involved in higher-level processing of emotional cognition and affective state, including representation of interpersonal relationships (37); interestingly, meta-analytic evidence in studies of patients with MDD/anxiety disorders indicates that psychotherapy such as CBT (but not conventional SSRIs) is typically associated with increased activity in this region(38). This differs from our findings with prucalopride in healthy volunteers, which found 5-HT_4_R agonism was associated with reduced activity in the medial pre-frontal cortex and the inferior parietal lobule during the same task. It is unclear whether this may be due to important differences between the compounds; while early studies with PF-04995274 characterised brain receptor occupancy (22), as it was developed with neurocognitive use in mind, this is less well-established for prucalopride which is known primarily for its effects on 5-HT_4_R in the gastrointestinal system (26,39), a system which may independently be relevant for depression (40). Alternatively, it may highlight the importance of translating findings into a clinical population; PET studies have already shown that the relationship between 5-HT_4_R function and cognitive and physiological domains (e.g. memory, cortisol awakening response) is reversed in healthy volunteers compared to participants with depression (17, 41, 42).

Participants receiving either active drug scored significantly lower on observer-rated depression severity at the end of the study compared to placebo. While somewhat unexpected considering the short duration of the study (7-9 days), early SSRI efficacy has been previously reported in some studies (e.g. 3,4). In the 5-HT_4_R agonist group, this was accompanied by significant reductions in self-reported depression, state anxiety and negative affect. The specific improvement in anxiety symptoms – also seen in analysis of the anxiety subscale of the HAM-D – is note-worthy given evidence that anxiety is a consistent predictor of worse clinical outcomes (e.g. 43, 44). While the citalopram group had significant unblinding, blinding was well-maintained in the 5-HT_4_R group. Interestingly, the citalopram group reported significant reductions in both negative and positive affect, perhaps suggestive of emotional blunting(45). Our fMRI finding of non-specific reduction in amygdala activation to emotional faces may correspond with this; however, the lack of amygdala differentiation between the valences of the faces across the whole group, as in our previous use of the task (26), may instead indicate that features of this version of the task (namely, more tightly cropped face images) reduce differentiation between emotional expressions.

Interpretation of clinical outcomes should be cautious; this study was not designed as a clinical efficacy trial, so depression severity was not our primary outcome and study duration was short. Exclusion criteria were also restrictive and participants had mild-moderate depression severity, so may not be representative of the patients prescribed antidepressants (which may have also limited the effect sizes). We did not collect baseline measures of emotion cognition, and so could not directly investigate the association between within-participant change in emotional cognition and change in clinical outcomes. Also, while we collected data on contraception use (due to findings indicating that oral hormonal contraception use reduces 5-HT4R binding (46)), and report averages for behavioural and clinical outcome measures split by hormonal contraception use (see Table S6), our sample was not powered to explore sub-group analyses. Nonetheless, we provide interesting preliminary experimental evidence in a clinical population that 5-HT_4_R agonism displays promise for antidepressant drug development. This is consistent with a recent emulated target trial from our group using large-scale USA electronic health records, which found that prescription of prucalopride is associated with reduced incidence of first ever depression, relative to other anti-constipation agents(46).

Collectively, these results suggest that any antidepressant efficacy of 5-HT_4_R agonism likely operates via mechanisms distinct from conventional SSRI antidepressants, and should be a focus for future research, particularly as this raises the possibility of complementary combination therapy. In our previous work with prucalopride, we reported pro-cognitive effects of 5-HT_4_R agonism on learning and memory tasks(26,39,48) and our finding of increased medial-frontal activity during emotional cognition may provide additional support for this. One speculation may be that 5-HT_4_R agonism indirectly improves mood by ameliorating cognitive impairment. Elsewhere, we report on additional results from the current study suggesting that 5-HT4 agonism also has therapeutic potential for treating cognitive symptoms of depression (25), however any causal link between cognitive improvement and overall clinical improvements remains unexplored.

In conclusion, this first randomised, experimental study in patients with depression found that 6-9 days of 5-HT_4_ R agonism with PF-04995274 produced neural and behavioural and neuroimaging effects that contrasted with those seen with conventional SSRI antidepressants, showing no reduction in negative bias but instead increased medial-frontal activity. Participants receiving the novel 5-HT_4_ R agonist also reported significantly lower depressive symptoms after 7-9 days, a finding that warrants confirmation in clinical trials and in populations with more severe symptomatology, alongside further experimental work to clarify the distinct underlying mechanisms.

## Supplementary Material

Supplementary Materials

## Figures and Tables

**Figure 3:**
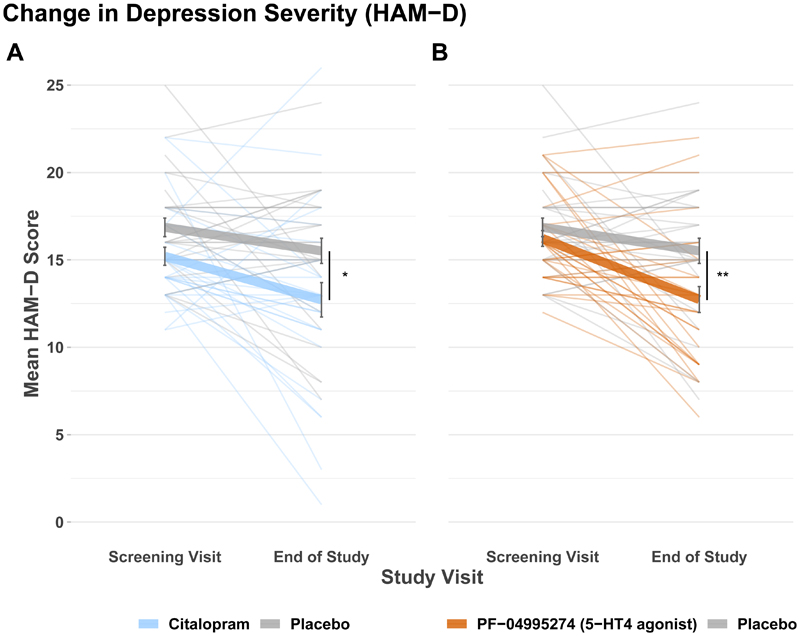
Participants given either PF-04995274 or citalopram displayed significantly reduced depressive symptoms on Hamilton Depression (HAM-D) Ratings after 7-9 days. HAM-D = Hamilton Depression Rating. **A** - citalopram group vs placebo group. **B** – PF-04995274 group vs placebo group. * = p < 0.05, ** = p< 0.01 for main effect of group on ANOVA, controlling for baseline score. Errors bars show standard error of the mean. Grey = placebo, Blue = citalopram, Orange = PF-04995274. Placebo group data is shown in both citalopram vs placebo plot (A), and in PF-04995274 vs placebo plots (B).

**Table 1:** Demographics and baseline self-report - All participants

Characteristic	5HT4, N = 31^1^	Citalopram, N = 30^1^	Placebo, N = 29^1^
Sex			
F	19 (61%)	19 (63%)	17 (59%)
M	12 (39%)	11 (37%)	12 (41%)
Age (decimal)	36.70 (13.10)	34.81 (12.06)	32.48 (10.33)
Ethnicity (grouped)			
White	27 (87%)	22 (73%)	24 (83%)
Other	4 (13%)	8 (27%)	5 (17%)
First language - English			
Y	19 (61%)	20 (67%)	23 (79%)
N	12 (39%)	10 (33%)	6 (21%)
Years of Education	16.71 (2.69)	16.90 (3.51)	17.30 (2.47)
BMI	24.97 (4.41)	25.15 (3.69)	25.51 (4.16)
Handedness - Edinburgh Inventory	18.76 (2.24)	17.43 (3.76)	18.03 (3.08)
Extraversion (EPQ)	8.52 (4.77)	8.93 (4.49)	9.07 (5.36)
Neuroticism (EPQ)	17.52 (4.25)	16.33 (4.18)	17.76 (4.14)
Psychoticism (EPQ)	4.45 (2.90)	4.00 (2.73)	3.38 (2.60)
Social desirability (EPQ)	7.58 (3.50)	7.30 (3.31)	8.31 (4.83)
Trait anxiety (STAIT)	58.65 (9.19)	57.77 (7.75)	59.31 (7.26)
Self report depressive symptoms (BDI)	24.16 (10.83)	24.45 (9.43)	24.86 (10.50)
Anhedonic symptoms (SHAPS)	4.32 (2.76)	3.83 (3.00)	3.83 (3.06)
Hormonal contraception (% of female participants)			
No hormonal contraception	13 (68%)	12 (63%)	9 (53%)
Oral contraceptive pill - hormonal	4 (21%)	1 (5.3%)	6 (35%)
Intra uterine system (IUS) - hormonal	1 (5.3%)	5 (26%)	0 (0%)
Unknown	1 (5.3%)	1 (5.3%)	2 (12%)

1 n (%); Mean (SD)

Ethnicity, other, self-defined: 5HT4 = Chinese, Eastern European, Askanazi Jewish; Citalopram = Asian (n=4), Black, Mixed, Mixed Indian and British; Placebo = Asian, Thai, Chinese, Mixed, Mixed Welsh/Indo guaynese

## Relevance Statement

Many patients with depression do not achieve satisfactory remission with SSRIs. This randomised experimental medicine study evaluated a novel 5-HT_4_R agonist, PF-04995274, in unmedicated patients with major depression. The agonist was associated with distinct brain activation patterns compared to a conventional SSRI (citalopram), and not with typical reductions in negative emotional bias. Although not designed as a clinical efficacy trial, the study also found improvement in depression symptoms after just one week. These findings point to a potential alternative antidepressant pathway and highlight the need for further mechanistic studies and clinical efficacy trials of 5-HT_4_R agonists.
