# Supplementary material for: Early effects of a novel 5-HT4 agonist (PF-04995274) and the SSRI citalopram on emotional cognition in unmedicated depression: the RESTAND study
Source: Br J Psychiatry. Author manuscript; Available in PMC 2026 Jul 21. (PMC7619257; doi:10.1192/bjp.2026.10664)
Supplement: Supplementary Materials [file EMS213437-supplement-Supplementary_Materials.pdf]

Early effects of a novel 5-HT_4 agonist (PF-04995274) and the SSRI citalopram on emotional cognition in unmedicated depression: the RESTAND study

SUPPLEMENTARY MATERIALS

Supplementary Methods

Complete inclusion criteria

- Male or female;
- Aged 18-65 years;
- Willing and able to give informed consent for participation in the study;
- Sufficiently fluent English to understand and complete the tasks;
- Registered with a GP and consents to GP being informed of participation in study;
- Meet DSM-V criteria for current Major Depressive Disorder [as determined by the Structured Clinical interview for DSM-V (SCID)];
- Participant must have received no drug or face-to-face psychological treatment for the current episode of depression/ in the previous six weeks;
- Participants engaging in sex with a risk of pregnancy must agree to use a highly effective method of contraception from Screening Visit until 30 days after receiving study medication treatment, female participants must not breastfeed, and male participants must not donate sperm.
  Acceptable methods of contraception include:
    - Combined (estrogen and progestogen containing) hormonal contraception associated with inhibition of ovulation; oral, intravaginal or transdermal
    - Progestogen-only hormonal contraception associated with inhibition of ovulation: oral, injectable or implantable
    - Intrauterine device (IUD)
    - Intrauterine hormone-releasing system (IUS)
    - Bilateral tubal occlusion
    - Vasectomy (or vasectomised partner)
    - Sexual abstinence. Periodic abstinence (calendar, symptothermal, post-ovulation methods), withdrawal (coitus interruptus), and spermicides only are not acceptable methods of contraception).

Complete exclusion criteria:

- History of or current DSM-V bipolar disorder, schizophrenia or eating disorders. Participants who fulfil current criteria for other comorbid disorders may still be entered into the study, if, in the opinion of the Investigator, the psychiatric diagnosis will not compromise safety or affect data quality;
- First-degree relative with a diagnosis of Bipolar Disorder type 1;
- Current usage of psychotropic medication;
- Failure to respond to antidepressant medication in current episode;
- Electroconvulsive therapy for the treatment of the current episode of depression;
- Participants undergoing any form of face-to-face structured psychological treatment during the study;
- Clinically significant abnormal values for liver function tests, clinical chemistry, urine drug screen, blood pressure measurement and ECG. A participant with a clinical abnormality or parameters outside the

reference range for the population being studied may be included only if the Investigator considers that the finding is unlikely to introduce additional risk factors and will not interfere with the study procedures;

- History of stimulant abuse (lifetime; e.g. amphetamine, cocaine), or of alcohol abuse within one year or of alcohol dependence within the lifetime;
- History of, or current medical conditions which in the opinion of the investigator may interfere with the safety of the participant or the scientific integrity of the study, including epilepsy/seizures, brain injury, severe hepatic or renal disease, severe gastro-intestinal problems, Central Nervous System (CNS) tumors, severe neurological problems (like Parkinson's; blackouts requiring hospitalisation);
- Medical conditions that may alter the hemodynamic parameters underlying the BOLD signal (e.g., inadequately treated hypertension, diabetes mellitus), if attending Research Visit One;
- Clinically significant risk of suicide;
- Current pregnancy (as determined by urine pregnancy test taken during Screening and First Dose visits), breastfeeding or planning a pregnancy during the course of the study;
- Participant not willing to use a suitable method of contraception for 30 days after receiving study drug treatment;
- Any contraindication to MRI scanning (e.g. metal objects in body, pacemakers, significant claustrophobia, pregnancy), if attending Research Visit One;
- Participants with Body Mass Index (BMI) outside the 18 to 36 kg/m2 range at the Screening Visit.
- Night-shift working or recent travel involving significant change of timezones;
- Excessive caffeine consumption, i.e., consumption higher than 8 cups of standard caffeinated drinks (tea, instant coffee) or higher than 6 cups of stronger coffee or other drinks containing methylxanthines such as coca cola or Red Bull per day;
- Participation in a psychological or medical study involving the use of medication within the last 3 months;
- Previous participation in a study using the same, or similar, emotional processing tasks;
- Smoker > 10 cigarettes per day or similar levels of tobacco consumption in other forms.
- Participant received prescribed medication within 28 days prior to Visit 2 (apart from the contraceptive pill). Participants who have taken prescription medication may still be entered into the study, if, in the opinion of the Investigator, the medication received will not interfere with the study procedures or compromise safety;
- Participant received non-prescription medication, including supplements such as vitamins and herbal supplements within 48 hours prior to Visit 2 (apart from paracetamol). Participants who have taken non-prescription medication may still be entered into the study, if, in the opinion of the Investigator, the medication received will not interfere with the study procedures or compromise safety;
- Participant with a known hypersensitivity to PF-04995274, citalopram or any other serotonergic agents;
- Participant with planned medical treatment within the study period that might interfere with the study procedures;
- Participant who is unlikely to comply with the clinical study protocol or is unsuitable for any other reason, in the opinion of the Investigator.
- Participant with Covid-19 symptoms or a household member with Covid-19 symptoms

Early effects of a novel 5-HT$_4$ agonist (PF-04995274) and the SSRI citalopram on emotional cognition in unmedicated depression: the RESTAND study

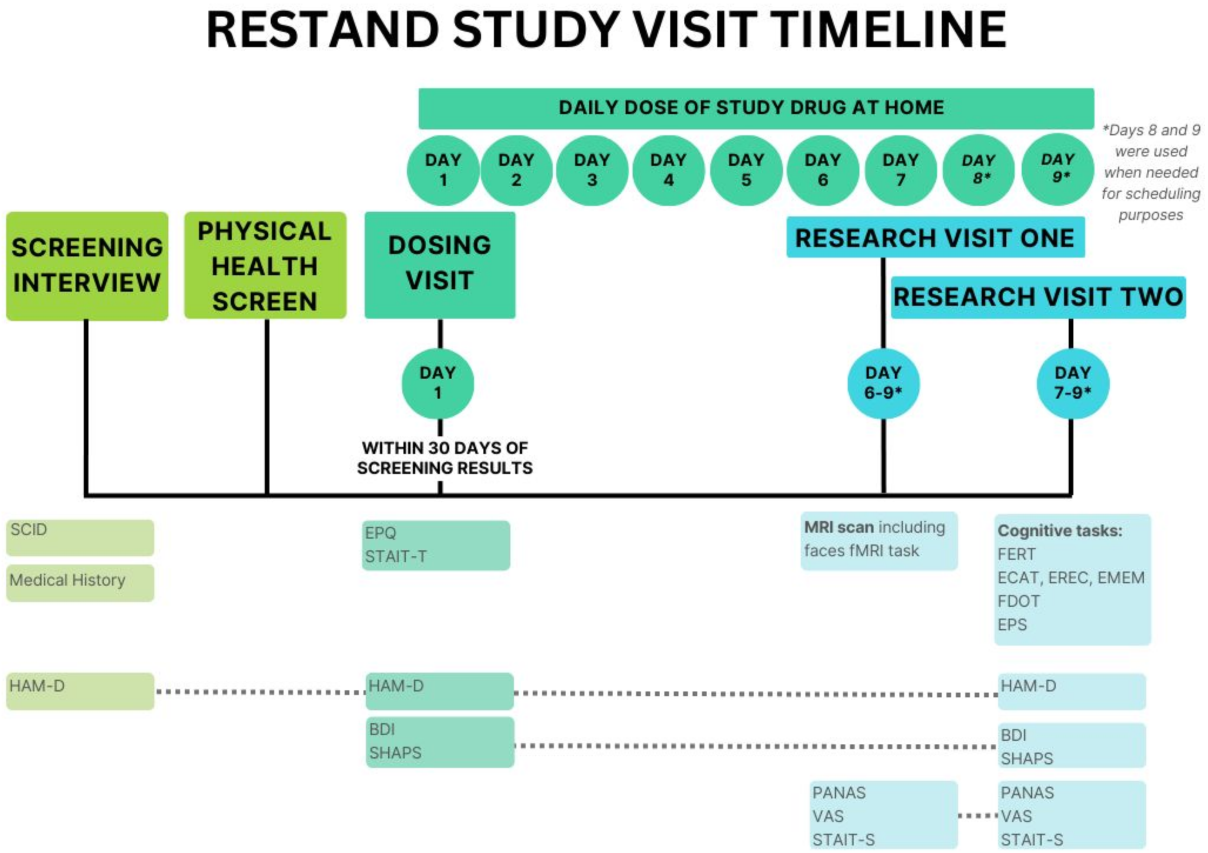

Figure S1. RESTAND Study Visit Timeline.

BDI = Beck Depression Inventory. ECAT = Emotional Categorisation Task. EMEM = Emotional Recognition Memory Task. EPQ = Eysenck Personality Questionnaire. EPS = Emotion Potentiated Startle task. EREC = Emotional Recall Task. FDOT = Faces Dot Probe Task. FERT = Facial Expression Recognition Task. HAM-D = Hamilton Depression Rating Scale, an observer-rated measure of depression severity. MRI = Magnetic Resonance Imaging. PANAS = Positive and Negative Affect Schedule. PILT = Probabilistic Learning Task. SCID = Structured Clinical Interview for DSM-5. SHAPS = Snaith-Hamilton Pleasure Scale. STAI-S = State Trait Anxiety Inventory, State version STAI-T = State Trait Anxiety Inventory, Trait version. VAS = Visual Analogue Scales.

Early effects of a novel 5-HT_4 agonist (PF-04995274) and the SSRI citalopram on emotional cognition in unmedicated depression: the RESTAND study

Description of additional emotional cognition tasks

Emotional Categorisation Task (ECAT): Negative (e.g. “domineering”, “untidy”, “hostile”) or positive (e.g. “cheerful”, “honest”, “optimistic”) personality descriptions are presented on screen and participants are asked to indicate whether they would like or dislike to be described as each of these characteristics, with a button-press.

Emotional Recall Task (EREC): Participants are asked to write down as many of the words as they can remember from the ECAT. Incorrect words are latter classified by two independent researchers as positively or negatively valenced.

Emotional Recognition Memory Task (EMEM): Participants are presented with positive (e.g. “domineering”, “untidy”, “hostile”) and negative (e.g. “cheerful”, “honest”, “optimistic”) personality describing words. They are asked to indicate for each word whether it was presented in the ECAT, with a button press.

Faces Dot Probe Task (FDOT): Two faces are presented vertically on the computer screen and are promptly followed by a pair of dots. The participant is asked to indicate whether the dots are vertically or horizontally aligned, with a button press. Some of the faces will have an emotional expression (e.g. fearful or happy).

Emotion potentiated startle (EPS): an electromyography (EMG) is used to measure eye-blink responses to a burst of white noise while participants are presented with pictorial stimuli that are either positively valenced, negatively valenced or neutral.

Probabilistic instrumental learning task (PILT), adapted from Pessiglione et al. (2006): Participants are asked to win as much money as possible, by selecting symbols associated with the highest probability of winning money and lowest probability of losing money. On each trial, participants are presented with two pairs of symbols: one pair is associated with win outcomes (win £0.20 or no change) and the other with loss outcomes (lose £0.20 or no change). Each symbol in the pair has reciprocal probabilities (70% or 30%) of either outcome occurring and is randomly positioned either to the left or the right of a central fixation cross.

Additional details about blinding

To achieve blinding between the three groups, each participant received 2 bottles, one containing 3 tablets and the other 1 capsule. Specifically, if they were assigned to the PF-04995274 treatment, they received 3 tablets of PF-04995274 (15mg in total) plus one capsule containing placebo. If they were assigned to the citalopram treatment, they received 1 encapsulated citalopram tablet (20 mg) plus 3 placebo tablets. Lastly, if participants were assigned to receive placebo, they received 3 placebo tablets plus 1 one capsule containing placebo.

Side effects checklist

SIDE EFFECT	ABSENT	MILD	MODERATE	SEVERE	WHEN & HOW LONG	STRENGTH OF BELIEF DUE TO DRUG (%)
SLEEPING PROBLEMS						
ABNORMAL DREAMING						
HEADACHE						
DIZZINESS						
SOMNOLENCE (feeling sleepy or sleeping a lot)						

Early effects of a novel 5-HT$_4$ agonist (PF-04995274) and the SSRI citalopram on emotional cognition in unmedicated depression: the RESTAND study

FEELING AGITATED						
FEELING ALERT						
NAUSEA (feeling sick)						
VOMITING (being sick)						
CONSTIPATION						
FATIGUE						
IMPULSE CONTROL PROBLEMS (strong urges to go shopping, have sex, spend money, gamble etc.)						
HALLUCINATIONS						
ABNORMAL MOVEMENTS						
DRY MOUTH						
OTHER (please specify:- ______________________________ ______________________________ ______________________________						

Data cleaning of emotion potentiated startle data

For emotion potentiated startle data, two researchers independently a) distinguished startle blink response from noise and decided whether a response could have been seen, had one occurred, excluding trials if a response could not be seen and b) determined if there was a blink response or if the trial should be recorded as a non-response. If there was disagreement, a third researcher made a final decision.

Additional details about fMRI data acquisition

fMRI data was acquired with a multiband echo-planar imaging (EPI) sequence of 72 T-2 weighted slices covering the whole brain (echo time (TE) = 30 ms; repetition time (TR) = 1200 ms; flip angle 65°, field of view 216 mm, slice thickness 2 mm, multiband acceleration factor 4, PAT (GRAPPA) factor, voxel dimension 2mm isotropic, acquisition time 6min 28s), along with an acquired map of the field distortions to correct for intensity bias (echos at 4.92 and 7.38 ms, TR=590ms, flip angle = 46°). Additional high-resolution T1-weighted structural scans were acquired using a gradient echo sequence (TR 1900ms, TE 3.97ms, flip angle 8°, field of view 192mm, voxel dimension 1 mm isotropic, acquisition time 5min 31s) to allow later registration of the fMRI data into standard space.

Additional details about fMRI pre-processing

Pre-processing involved various steps designed to reduce noise-related variability in the data and to improve the validity of the statistical analysis. Each participant's functional imaging data underwent the following steps: (1) Removal of non-brain structures using BET, (2) motion correction using MCFLIRT, (3) spatial smoothing using a Gaussian kernel of FWHM 5 mm, (4) grand-mean intensity normalisation of the entire 4D dataset by a single multiplicative factor and high-pass temporal filtering cut-off = 90 s (Gaussian-weighted least-squares straight-line fitting, with sigma = 45 s), and (5) B0 unwarping using fieldmap rads and magnitude images for distortion correction.

Early effects of a novel 5-HT_4 agonist (PF-04995274) and the SSRI citalopram on emotional cognition in unmedicated depression: the RESTAND study

Outlier on behavioural emotional cognition task

The outlier participant was in the citalopram group. They were deemed an outlier because their average reaction time was 4124ms - all other participants fell between 1042 and 2378ms. A plot of reaction time distributions is below.

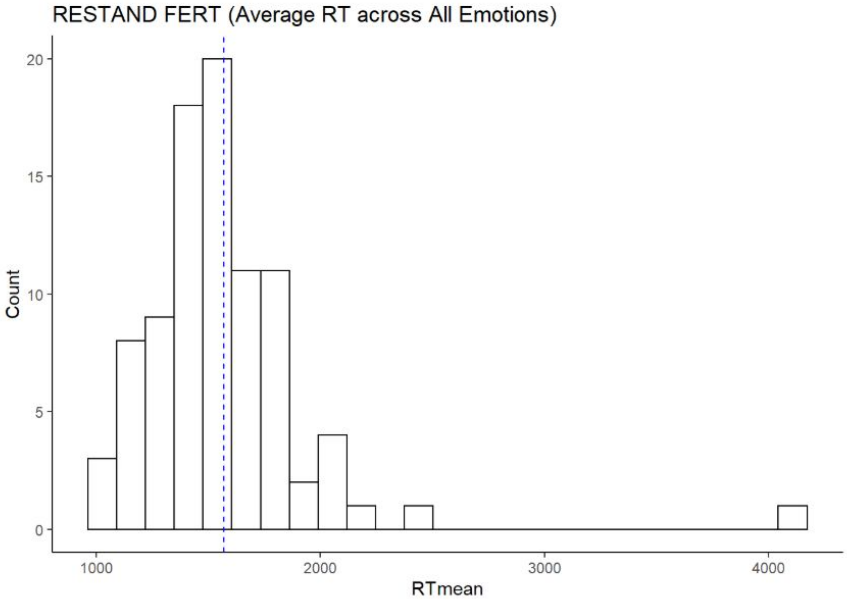

All reported stats for citalopram vs placebo in the manuscript include this participant. They were excluded for sensitivity checks, to ensure they didn't have a notable effect on the results, and in these sensitivity checks the significance and direction of effect remained the same:

	All citalopram vs placebo participants	Without citalopram outlier
Accuracy	Main effect<br>(F(1,56)=0.49, p=0.49, ηp2=0.009)<br><br>Interaction<br>(F(1,56)=4.30, p=0.043, ηp2 =0.07).	Main effect<br>(F(1,55)=0.366, p=0.55, ηp2=0.007)<br><br>Interaction<br>(F(1,55)=4.50, p=0.038, ηp2 =0.08).
Reaction time	Main effect<br>(F(1,56)=0.98, p=0.325, ηp2=0.02)<br><br>Interaction<br>(F(1,56)=6.78, p=0.012, ηp2=0.11)	Main effect<br>(F(1,55)=0.151, p=0.699, ηp2=0.03)<br><br>Interaction<br>(F(1,55)=6.67, p=0.013, ηp2=0.11)

Early effects of a novel 5-HT_4 agonist (PF-04995274) and the SSRI citalopram on emotional cognition in unmedicated depression: the RESTAND study

Supplementary Results

Comorbidity data – Table S1

Medication group	Comorbidities
PF-04995274	Anxiety disorder – 12/31<br>PTSD symptoms in past or not meeting criteria – 2/31<br>None reported – 14/31<br>Missing – 4/31
Citalopram	Anxiety disorder – 10/30<br>Other depressive disorder 1/30<br>None reported 11/30<br>Missing – 8/30
Placebo	Anxiety disorder –14/29<br>Depersonalisation – 1/29<br>None reported – 9/29<br>Missing – 5/29

FERT

Misclassification rates

When exploring the misclassification rates i.e. whether medication group was associated with differences in misclassification of faces as positive, neutral, or negative emotions, there were no significant associations but a sensitivity analysis (potential outlier removal) indicated a trend for the citalopram group having a reduced negative misclassification rate compared to placebo ($F(1,55)=3.056$, $p=0.086$). There was no significant difference between citalopram and placebo group in positive or neutral misclassification rate ($p>0.12$).

When comparing the PF-04995274 group to placebo, there was no significant difference in negative, neutral or positive misclassification rate ($p>0.4$).

ECAT, EREC, and EMEM

On the ECAT, EREC and EMEM, when comparing citalopram to placebo, there was no main effect of medication group (ECAT accuracy, $F(1,112)= 1.308$, $p =0.255$, $\eta p2=0.01$; ECAT reaction time, $F(1,112) = 0.782$, $p=0.378$, $\eta p2=0.007$; EREC correct words recalled, $F(1,114)= 0.091$, $p =0.763$, $\eta p2=0.0008$; EREC incorrect words recalled, $F(1,114)= 0.267$, $p =0.606$, $\eta p2=0.002$; EMEM total hits, $F(1,110)=0.003$, $p=0.955$, $\eta p2=0.00003$; EMEM total correct rejections, $F(1,110)=0.928$, $p=0.33747$, $\eta p2=0.008$; EMEM reaction time, $F(1,110)=0.040$, $p=0.841$, $\eta p2=0.0004$).

There was also no interaction between valence and medication group (ECAT accuracy, $F(1,112)= 1.574$, $p =0.212$, $\eta p2=0.01$; ECAT reaction time, $F(1,112) = 0.150$, $p=0.699$, $\eta p2=0.001$; EREC correct words recalled, $F(1,114)= 0.184$, $p=0.668$, $\eta p2=0.002$; EREC incorrect words recalled, $F(1,114)= 0.002$, $p =0.967$, $\eta p2=0.00001$; EMEM total hits, $F(1,110)=0.031$, $p=0.860$, $\eta p2=0.0003$; EMEM total correct rejections, $F(1,110)=0.285$, $p=0.59483$, $\eta p2=0.003$; EMEM reaction time, $F(1,110)=0.058$, $p=0.811$, $\eta p2=0.0005$).

Similarly, when comparing the PF-04995274 group to placebo, there was no main effect of medication group (ECAT accuracy, $F(1,114)= 0.009$, $p =0.924$, $\eta p2=0.00008$; ECAT reaction time, $F(1,114) =$

0.002, p=0.966, ηp2=0.00002; EREC incorrect words recalled, F(1,116)= 0.259, p =0.612, ηp2=0.002; EMEM total hits, F(1,114)=1.062, p=0.305, ηp2=0.009; EMEM total correct rejections, F(1,114)=0.240, p=0.62517, ηp2=0.002; EMEM reaction time, F(1,114)=0.158, p=0.692, ηp2=0.001); the only exception was a main effect of medication group on total correct words recalled in the EREC, with the PF-04995274 group recalling fewer words (F(1,116)= 5.321, p =0.0228, ηp2=0.04).

There was also no interaction between valence and medication group (ECAT accuracy, F(1,114)= 0.006, p =0.936, ηp2=0.00006; ECAT reaction time, F(1,114) = 0.221, p=0.639, ηp2=0.002; EREC correct words recalled, F(1,116)= 0.005, p =0.944, ηp2=0.00004; EREC incorrect words recalled, F(1,116)= 0.566, p =0.453, ηp2=0.005; EMEM total hits, F(1,114)=0.106, p=0.746, ηp2=0.0009; EMEM total correct rejections, F(1,114)=0.581, p=0.447, ηp2=0.005; EMEM reaction time, F(1,114)=0.072, p=0.790, ηp2=0.0006).

All results remained the same in sensitivity analyses, except that a main effect of medication group on EMEM total hits became significant, with the PF-04995274 group identifying less hits than the placebo group (F(1,108)=5.078, p=0.0263, ηp2=0.04).

FDOT

There was no main effect of valence or mask. Neither the citalopram group nor the PF-04995274 group showed a significant main effect of medication (citalopram vs placebo, F(1,224)=0.052, p=0.820, ηp2=0.0002; PF-04995274 vs placebo, F(1,228)=0.949, p=0.331, ηp2=0.004), an interaction between medication group and valence (citalopram vs placebo, F(1,224)=0.149, p=0.700, ηp2=0.0007; PF-04995274 vs placebo, F(1,228)=0.864, p=0.354, ηp2=0.004), or an interaction between medication group, valence and mask (citalopram vs placebo, F(1,224)=1.809, p=0.180, ηp2=0.008; PF-04995274 vs placebo, F(1,228)=0.003, p=0.953, ηp2=0.00002). The results of these analyses did not change in sensitivity analyses.

EPS

Across the whole sample, there was no evidence of the expected emotion potentiation – the main effect of image valence was non-significant (p>0.15) for amplitude (raw or Z scored VMax) and latency (TMax, p>0.75), and the largest amplitudes were seen for neutral stimuli. This pattern and lack of significance remained true within the placebo group as well.

When comparing citalopram to placebo on raw amplitude, there was a main effect of valence (F(2, 21)=3.76, p=0.040, ηp2=0.26), with mean amplitude for neutral being highest, followed by negative and then positive valence. There was also an interaction between group and valence (F(2,21)=4.08, p= 0.032, ηp2=0.28), with those on citalopram having greater amplitudes than placebo for neutral and negative images, and reduced amplitudes for positive images. In sensitivity analyses, the main effect of emotion became non-significant (F(2,20)= 0.99, p=0.39, ηp2=0.09), but the interaction remained (F(2,20)= 3.55, p=0.048, ηp2=0.26). When comparing PF-04995274 to placebo on raw amplitude, there was no significant interaction between group and valence (p>0.18).

Early effects of a novel 5-HT_4 agonist (PF-04995274) and the SSRI citalopram on emotional cognition in unmedicated depression: the RESTAND study

When comparing citalopram to placebo on z-scored amplitude, there was no main effect of valence or interaction between group and valence ($p>0.12$). The same was true when comparing PF-04995274 to placebo ($p>0.15$).

When comparing citalopram to placebo on latency, there was a main effect of group ($F(1,21)=12.36$, $p=0.002$, $\eta p2=0.37$), with citalopram showing shorter latency, but no interaction with valence ($F(2,21)=0.202$, $p=0.82$, $\eta p2=0.02$). This remained true in sensitivity analyses. When comparing PF-04995274 to placebo on latency, there was no main effect of group or interaction between group and valence ($p>0.2$).

PILT

Neither the citalopram group nor the PF-04995274 group showed a significant main effect of medication on choice of optimal symbol in win trials (citalopram vs placebo, $F(1,53)=1.006$, $p=0.32$, $\eta p2=0.02$; PF-04995274 vs placebo, $F(1,54)=0.095$, $p=0.759$, $\eta p2=0.002$), or in loss trials (citalopram vs placebo, $F(1,53)=0.451$, $p=0.505$, $\eta p2=0.008$; PF-04995274 vs placebo, $F(1,54)=0.01$, $p=0.922$, $\eta p2=0.0002$). The results of these analyses did not change in sensitivity analyses.

fMRI behavioural results

Overall, the mean percentage accuracy for gender classification was 85.6% (sd=7.09, range 65.48-95.24%). For both groups, there was no main effect of group or interaction between group and valence, on percentage accuracy (citalopram vs placebo, main effect $F(1,92)=1.126$, $p=0.219$, $\eta p2=0.01$, interaction $F(1,92)=0.017$, $p=0.896$, $\eta p2=0.0001$; PF-04995274 vs placebo, main effect $F(1,100)=0.821$, $p=0.367$, $\eta p2=0.008$, interaction $F(1,100)=0.357$, $p=0.552$, $\eta p2=0.003$).

Overall, the mean reaction time was 642ms (sd=13, range 441-916ms). When comparing the citalopram and placebo groups, there was a significant main effect of medication group on reaction time ($F(1,92)=12.914$, $p=0.0005$, $\eta p2=0.12$), with the citalopram group showing faster mean reaction times than placebo (m=603ms, sd=12.2 vs m=683ms, sd=9.8) but no interaction with valence of face ($F(1,92)=0.821$, $p=0.367$, $\eta p2=0.008$). When comparing the PF-04995274 group to placebo, there was no significant main effect of medication ($F(1,100)=2.981$, $p=0.087$, $\eta p2=0.03$), and no interaction between medication group and valence ($F(1,100)=0.197$, $p=0.658$ $\eta p2=0.02$).

fMRI data - task effects

When comparing mean activation to emotional faces (across fearful and happy faces) to activation during fixation in whole brain analysis, across all groups, there were significant clusters covering occipital pole, right cerebral cortex, post central gyrus, angular gyrus, precentral gyrus, cingulate gyrus, para-cingulate gyrus, left and right putamen, insular cortex, left thalamus, and left and right amygdala. ROI analyses found consistent activation in both amygdala, orbitofrontal cortex and medial-frontal cortex. These results are consistent with previous reports using the same task, suggesting that the task was successful in probing emotional processing. When comparing fear to happy i.e. looking at differential activation related to face valence, there were three significant clusters

for fear > happy, two in cerebellum and one in the fusiform gyrus, and six significant clusters for fear < happy, including paracingulate gyrus, fusiform gyrus, middle and superior frontal gyrus, and brain stem. In ROI analyses, there was no significant differential activation of either amygdala, or the orbitofrontal cortex, but there was consistent significant activation associated with happy>fear in medial-frontal cortex.

fMRI whole brain analyses

Early effects of a novel 5-HT₄ agonist (PF-04995274) and the SSRI citalopram on emotional cognition in unmedicated depression: the RESTAND study

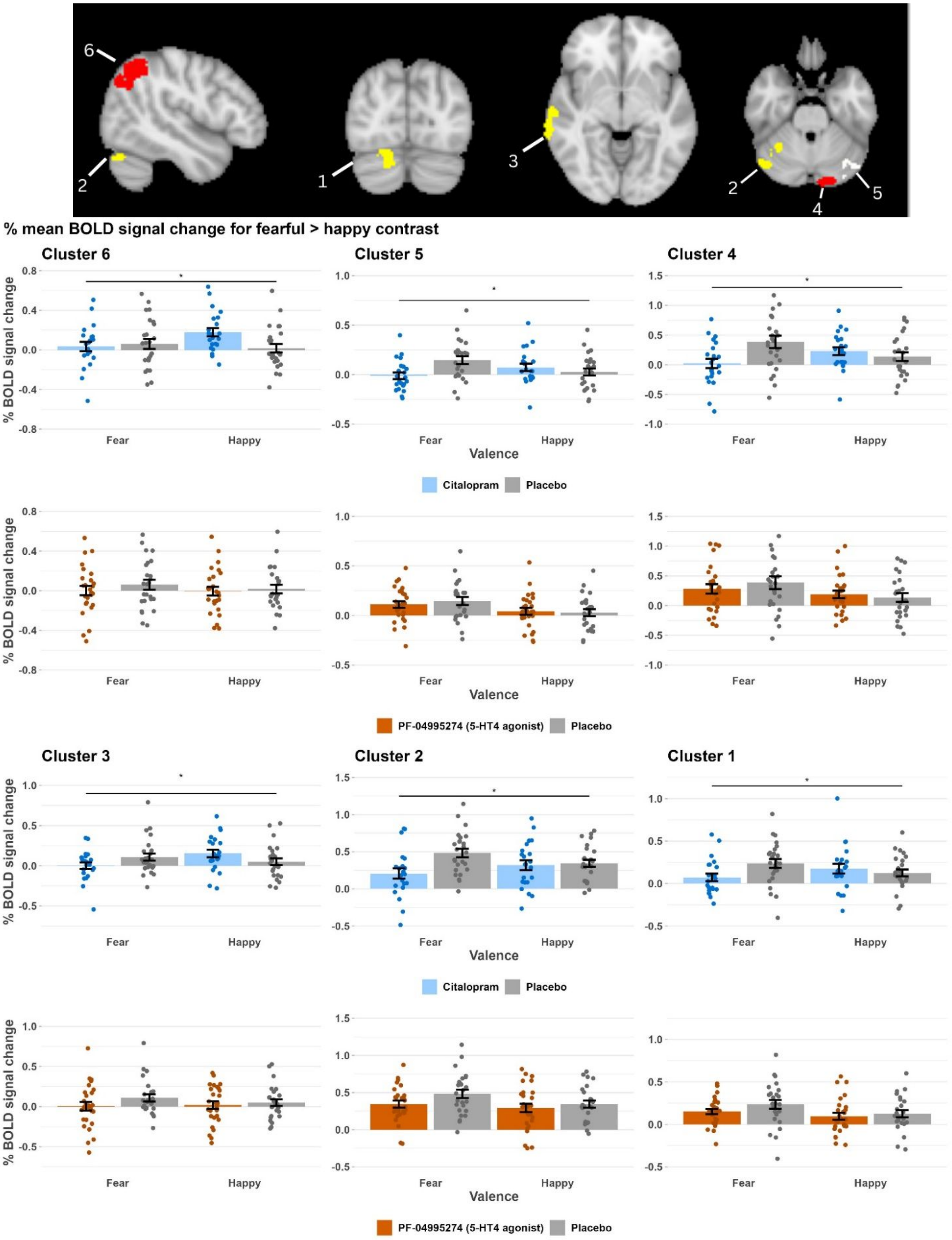

Figure S2. % mean BOLD signal change in 6 clusters with a significant difference between citalopram and placebo group for fearful > happy contrast. *Cluster 1 = occipital fusiform gyrus, lingual gyrus. Cluster 2 = cerebellum, occipital fusiform gyrus, lateral occipital. Cluster 3 = middle temporal gyrus (posterior), superior temporal gyrus. Cluster 4 = cerebellum, occipital fusiform, cerebral cortex. Cluster 5 = cerebellum. Cluster 6 = angular gyrus and lateral occipital.*

** = p < 0.05, ** = p < 0.01 for interaction between group and valence on ANOVA. Errors bars show standard error of the mean. Grey = placebo, Blue = citalopram, Orange = PF-04995274.*

Early effects of a novel 5-HT$_4$ agonist (PF-04995274) and the SSRI citalopram on emotional cognition in unmedicated depression: the RESTAND study – SUPPLEMENTARY MATERIALS

Table S2

Cluster number	Size (voxels)	F statistic (valence * contrast)	p-value	Z-max location (MNI)	Main regions involved in cluster	Mean fear – mean happy BOLD signal change %
6	541	Citalopram vs. placebo – F(1,90)= 4.101<br>PF-04995274 vs. placebo – F(1,100) = 0.170	0.046<br>0.681	48, 50, 46,	Angular gyrus and lateral occipital	Placebo =– 0.043<br>Citalopram= -0.143<br>PF-04995274 = 0.005
5	466	Citalopram vs. placebo – F(1,90)= 7.604<br>PF-04995274 vs. placebo – F(1,100) = 0.482	0.007<br>0.489	-40, -68, -48	Cerebellum	Placebo = 0.118<br>Citalopram = -0.085<br>PF-04995274 = 0.069
4	393	Citalopram vs. placebo – F(1,90)= 7.211<br>PF-04995274 vs. placebo – F(1,100) = 0.932	0.009<br>0.337	-20, -86, -28	Cerebellum, occipital fusiform, cerebral cortex	Placebo = 0.249<br>Citalopram = -0.206<br>PF-04995274 = 0.092
3	279	Citalopram vs. placebo – F(1,90)= 5.695<br>PF-04995274 vs. placebo – F(1,100) = 0.597	0.019<br>0.441	68, -36, -8	Middle temporal gyrus (posterior), superior temporal gyrus	Placebo = 0.058<br>Citalopram = -0.152<br>PF-04995274 = -0.016
2	191	Citalopram vs. placebo – F(1,90)= 4.408<br>PF-04995274 vs. placebo – F(1,100) = 0.679	0.039<br>0.412	40, -70, -26	Cerebellum, occipital fusiform, lateral occipital	Placebo = 0.138<br>Citalopram = -0.113<br>PF-04995274 = 0.052
1	116	Citalopram vs. placebo – F(1,90)= 4.71<br>PF-04995274 vs. placebo – F(1,100) = 0.436	0.033<br>0.511	16, -82, -20	Occipital fusiform, lingual gyrus	Placebo = 0.111<br>Citalopram = -0.105<br>PF-04995274 = 0.055

HAM-D subscales and individual items – Table S3

HAM-D Item (s)	PF-04995274, N = 31[1]	Citalopram, N = 30[1]	Placebo, N = 29[1]	F statistic	P value	Partial eta squared (ηp2)
Core six symptoms only (items 1,2,7,8,10,13)	Screening<br>9.84 (2.13)<br>End of study<br>7.7 (3.00)	Screening<br>9.43 (1.48)<br>End of study<br>8.15 (2.93)	Screening<br>10.5 (1.76)<br>End of study<br>9.76 (2.80)	**Citalopram vs. placebo - F(1,53)=5.53**<br>**PF-04995274 vs. placebo - F(1,56)=9.99**	**0.0225**<br>**0.0025**	0.09<br>0.15
All items, excluding sleep (exc 4,5 6)	Screening<br>13.7 (2.5)<br>End of study<br>10.6 (4.23)	Screening<br>11.6 (3.66)<br>End of study<br>9.97 (5.19)	Screening<br>14.0 (2.29)<br>End of study<br>13.3 (4.37)	**Citalopram vs. placebo - F(1,56)=9.38**<br>**PF-04995274 vs. placebo - F(1,57)=7.089**	**0.0034**<br>**0.0101**	0.14<br>0.11
Anxiety factor (items 10, 11, 12, 13, 15, 17)	Screening<br>5.19 (1.42)<br>End of study<br>4.07 (2.26)	Screening<br>4.75 (1.43)<br>End of study<br>4.44 (2.06)	Screening<br>5.62 (1.47)<br>End of study<br>5.69 (2.93)	Citalopram vs. placebo - F(1,53)= 3.346<br>**PF-04995274 vs. placebo - F(1,56)=6.576**	0.073<br>**0.0131**	0.06<br>0.11
Item 1 only – low mood	Screening<br>2.52 (0.724)<br>End of study<br>1.9 (1.03)	Screening<br>2.36 (0.559)<br>End of study<br>1.82 (0.921)	Screening<br>2.55 (0.632)<br>End of study<br>2.14 (0.875)			
Item 2 only – feelings of guilt	Screening<br>1.13 (0.806)<br>End of study<br>1 (0.83)	Screening<br>1.04 (0.637)<br>End of study<br>0.778 (0.641)	Screening<br>1.28 (0.455)<br>End of study<br>1.14 (0.581)			
Item 3 only - suicide	Screening<br>0.387 (0.495)<br>End of study<br>0.3 (0.535)	Screening<br>0.286 (0.46)<br>End of study<br>0.222 (0.506)	Screening<br>0.31 (0.541)<br>End of study<br>0.31 (0.541)			

Early effects of a novel 5-HT$_4$ agonist (PF-04995274) and the SSRI citalopram on emotional cognition in unmedicated depression: the RESTAND study – SUPPLEMENTARY MATERIALS

Item 4 only – insomnia early in night	Screening<br>0.871 (0.806)<br><br>End of study<br>0.567 (0.679)	Screening<br>0.929 (0.858)<br><br>End of study<br>0.63 (0.792)	Screening<br>1.14 (0.915)<br><br>End of study<br>0.931 (0.884)	
Item 5 only – insomnia, middle of night	Screening<br>0.903 (0.79)<br><br>End of study<br>0.767 (0.817)	Screening<br>0.857 (0.705)<br><br>End of study<br>0.815 (0.834)	Screening<br>0.862 (0.743)<br><br>End of study<br>0.724 (0.751)	
Item 6 only – insomnia, early morning	Screening<br>0.839 (0.779)<br><br>End of study<br>0.767 (0.774)	Screening<br>0.786 (0.876)<br><br>End of study<br>0.519 (0.849)	Screening<br>0.793 (0.675)<br><br>End of study<br>0.724 (0.797)	
Item 7 only – work and activities	Screening<br>2.32 (0.653)<br><br>End of study<br>1.77 (0.817)	Screening<br>2.43 (0.879)<br><br>End of study<br>2.18 (1.04)	Screening<br>2.31 (0.761)<br><br>End of study<br>2.07 (0.842)	
Item 8 only – psychomotor retardation	Screening<br>0.677 (0.541)<br><br>End of study<br>0.6 (0.498)	Screening<br>0.536 (0.508)<br><br>End of study<br>0.444 (0.506)	Screening<br>0.793 (0.675)<br><br>End of study<br>0.828 (0.759)	
Item 9 only - agitation	Screening<br>0.806 (0.946)<br><br>End of study<br>0.733 (0.785)	Screening<br>0.5 (0.743)<br><br>End of study<br>0.481 (0.509)	Screening<br>0.655 (0.857)<br><br>End of study<br>0.69 (0.85)	
Item 10 only – anxiety psychological	Screening<br>1.48 (0.769)<br><br>End of study<br>1.2 (0.887)	Screening<br>1.39 (0.737)<br><br>End of study<br>1.48 (1.01)	Screening<br>1.79 (0.94)<br><br>End of study<br>1.93 (1.07)	
Item 11 only – anxiety somatic	Screening<br>1.1 (0.746)<br><br>End of study<br>0.833 (0.791)	Screening<br>1.11 (0.875)<br><br>End of study<br>0.815 (0.834)	Screening<br>1.34 (0.897)<br><br>End of study<br>1.14 (0.953)	
Item 12 only – somatic gastrointestinal	Screening<br>0.645 (0.661)<br><br>End of study<br>0.433 (0.626)	Screening<br>0.321 (0.476)<br><br>End of study<br>0.481 (0.7)	Screening<br>0.517 (0.574)<br><br>End of study<br>0.379 (0.494)	
Item 13 only – general somatic	Screening<br>1.71 (0.529)<br><br>End of study<br>1.23 (0.817)	Screening<br>1.68 (0.548)<br><br>End of study<br>1.44 (0.698)	Screening<br>1.76 (0.511)<br><br>End of study<br>1.66 (0.67)	
Item 14 only – genital	Screening<br>0.581 (0.72)<br><br>End of study<br>0.467 (0.571)	Screening<br>0.464 (0.744)<br><br>End of study<br>0.63 (0.839)	Screening<br>0.448 (0.527)<br><br>End of study<br>0.4498(0.527)	
Item 15 only - hypochondriasis	Screening<br>0.161 (0.454)<br><br>End of study<br>0.233 (0.504)	Screening<br>0.179 (0.548)<br><br>End of study<br>0.074 (0.267)	Screening<br>0.103 (0.409)<br><br>End of study<br>0.483 (1.7)	
Item 16 only – weight loss	Screening<br>0.129 (0.428)<br><br>End of study<br>0.1 (0.305)	Screening<br>0.071 (0.262)<br><br>End of study<br>0.074 (0.267)	Screening<br>0.069 (0.258)<br><br>End of study<br>0..034 (0.186)	
Item 17 only - insight	Screening<br>0.097 (0.301)<br><br>End of study<br>0.133 (0.346)	Screening<br>0.071 (0.262)<br><br>End of study<br>0.148 (0.456)	Screening<br>0.103 (0.31)<br><br>End of study<br>0.103 (0.31)	

Early effects of a novel 5-HT$_4$ agonist (PF-04995274) and the SSRI citalopram on emotional cognition in unmedicated depression: the RESTAND study – SUPPLEMENTARY MATERIALS

End of study self-report – Table S4

Characteristic	PF-04995274, N = 31[1]	Citalopram, N = 30[1]	Placebo, N = 29[1]	F statistic	P value	Partial eta squared (ηp2)
Depressive symptoms (BDI)[+]	**17.69 (11.38)**	20.63 (10.82)	**22.38 (9.28)**	Citalopram vs. placebo - F(1,52)=0.44<br>**PF-04995274 vs. placebo** - F(1,55)=6.33	0.5<br>**0.015**	0.008<br>0.1
Anhedonic symptoms (SHAPS)[+]	3.31 (2.71)	3.93 (2.42)	3.45 (2.81)	Citalopram vs. placebo – F(1,52)=0.87<br>PF-04995274 vs. placebo - F(1,54)=0.05	0.36<br>0.82	0.02<br>0.001
State anxiety across final research visit (STAI-S)	**42.46 (9.70)**	44.17 (10.82)	**45.01 (9.77)**	Citalopram vs. placebo - F(1,267)=0.31<br>**PF-04995274 vs. placebo** - F(1,273)= 3.92	0.58<br>**0.049**	0.001<br>0.02
Positive affect across final research visit (PANAS - P)	21.47 (7.35)	**20.18 (5.14)**	**22.03 (6.63)**	**Citalopram vs. placebo** – F(1,267)=7.29<br>PF-04995274 vs. placebo - F(1,273)=0.42	**0.007**<br>0.52	0.02<br>0.002
Negative affect across final research visit (PANAS - N)	**14.80 (4.80)**	**15.54 (5.60)**	**16.99 (5.82)**	**Citalopram vs. placebo** - F(1,267)=4.93<br>**PF-04995274 vs. placebo** - F(1,273)=10.75	**0.027**<br>**0.001**	0.02<br>0.04

[1]Mean (SD). BDI = Beck's Depression Inventory. PANAS = Positive and Negative Affect Schedule. P = Positive subscale. N = Negative subscale. SHAPS = Snaith-Hamilton Pleasure Scale. STAI-S = State Trait Anxiety Inventory. [+]HAM-D and BDI analyses controlled for baseline severity.

Self-reported affect – Table S5

Early effects of a novel 5-HT4 agonist (PF-04995274) and the SSRI citalopram on emotional cognition in unmedicated depression: the RESTAND study – SUPPLEMENTARY MATERIALS

**Visual Analogue Scale**	**PF-04995274**, N = 31[1]	**Citalopram**, N = 30[1]	**Placebo**, N = 29[1]	**F statistic**	**P value**	**Partial eta squared (ηp2)**
Current happiness	46.97 (18.39)	43.27 (18.03)	45.31 (16.78)	Citalopram vs. placebo – F(1,57)= 0.202<br>PF-04995274 vs. placebo – F(1,58) =0.133	0.66<br>0.72	0.004<br>0.002
Current sadness	29.72 (24.09)	35.16 (24.61)	34.14 (18.65)	Citalopram vs. placebo - F(1,57)= 0.033<br>PF-04995274 vs. placebo - F(1,58)= 0.623	0.86<br>0.43	0.0006<br>0.01
Current hostility	9.34 (13.17)	11.57 (16.84)	11.55 (12.90)	Citalopram vs. placebo - F(1,57)= 0.00<br>PF-04995274 vs. placebo - F(1,58)= 0.431	0.99<br>0.51	<0.0001<br>0.007
Current alertness	35.30 (18.57)	39.98 (19.31)	44.16 (18.95)	Citalopram vs. placebo - F(1,57)=0.71<br>PF-04995274 vs. placebo - F(1,58)=3.35	0.404<br>0.07	0.01<br>0.05
Current anxiety	29.05 (26.11)	33.67 (25.97)	38.60 (21.81)	Citalopram vs. placebo – F(1,57)=0.62<br>PF-04995274 vs. placebo - F(1,58)=2.34	0.43<br>0.13	0.01<br>0.04
Current calmness	49.51 (18.32)	51.23 (19.25)	50.81 (19.45)	Citalopram vs. placebo – F(1,58)=0.007<br>PF-04995274 vs. placebo – F(1,58)=0.071	0.93<br>0.791	0.0001<br>0.0012
[1]*Mean, averaged across final research visit measures (SD)*						

Side-effects – Table S6

**Reports of Side Effect**		**PF-04995274**, N = 31[1]	**Citalopram**, N = 28[1]	**Placebo**, N = 28[1]	**Group difference in number of participants who reported**
**Abdominal pain**	During dosing reports	1 (3.2%)	0 (0%)	0 (0%)	
	At home reports	2 (6.5%)	1 (3.6%)	3 (11%)	
	Final visit reports	3 (9.7%)	0 (0%)	3 (11%)	
	**Overall participants reporting**	**5**	**1**	**5**	**X^2=2.91, df=2, p=.23**
**Constipation**	During dosing reports	0 (0%)	0 (0%)	0 (0%)	
	At home reports	1 (3.2%)	2 (7.1%)	0 (0%)	
	Final visit reports	0 (0%)	3 (11%)	1 (3.6%)	

Side-effects – Table S6

Reports of Side Effect		PF-04995274, N = 31[1]	Citalopram, N = 28[1]	Placebo, N = 28[1]	Group difference in number of participants who reported
	**Overall participants reporting**	**1**	**4**	**1**	**X²=3, df=2, p=.22**
**Diarrhea***	At home reports	3 (9.7%)	3 (11%)	2 (7.1%)	NA*
	Final visit reports	1 (3.2%)	0 (0%)	0 (0%)	
**Increased appetite***	At home reports	3 (9.7%)	0 (0%)	0 (0%)	NA*
	Final visit reports	1 (3.2%)	0 (0%)	0 (0%)	
**Nausea**	During dosing reports	0 (0%)	3 (11%)	0 (0%)	
	At home reports	3 (9.7%)	4 (14%)	2 (7.1%)	
	Final visit reports	5 (16%)	2 (7.1%)	4 (14%)	
	**Overall participants reporting**	**7**	**6**	**4**	**X²=0.82, df=2, p=.66**
**Vomiting**	During dosing reports	0 (0%)	1 (3.6%)	0 (0%)	NA – numbers too small
	At home reports	0 (0%)	0 (0%)	0 (0%)	
	Final visit reports	0 (0%)	0 (0%)	0 (0%)	
**Other gastrointestinal side effect***	During dosing reports	1 (3.2%)	0 (0%)	2 (7.1%)	NA*
	At home reports	0 (0%)	2 (7.1%)	1 (3.6%)	
	Final visit reports	0 (0%)	1 (3.6%)	1 (3.6%)	
**Abnormal dreaming**	During dosing reports	0 (0%)	0 (0%)	0 (0%)	
	At home reports	1 (3.2%)	1 (3.6%)	3 (11%)	
	Final visit reports	3 (9.7%)	3 (11%)	1 (3.6%)	
	**Overall participants reporting**	**4**	**3**	**3**	**X²=0.2, df=2, p=.91**
**Fatigue**	During dosing reports	2 (6.5%)	0 (0%)	1 (3.6%)	
	At home reports	4 (13%)	2 (7.1%)	1 (3.6%)	
	Final visit reports	3 (9.7%)	2 (7.1%)	1 (3.6%)	
	**Overall participants reporting**	**8**	**3**	**1**	**X²=6.5, df=2, p=.04****
**Sleeping problems**	During dosing reports	1 (3.2%)	0 (0%)	2 (7.1%)	
	At home reports	1 (3.2%)	5 (18%)	0 (0%)	
	Final visit reports	2 (6.5%)	5 (18%)	3 (11%)	
	**Overall participants reporting**	**3**	**8**	**4**	**X²=2.8, df=2, p=.25**
**Sleepiness or sleeping a lot**	During dosing reports	2 (6.5%)	5 (18%)	2 (7.1%)	
	At home reports	2 (6.5%)	5 (18%)	4 (14%)	
	Final visit reports	2 (6.5%)	9 (32%)	5 (18%)	
	**Overall participants reporting**	**4**	**10**	**7**	**X²=2.57, df=2, p=.28**
**Abnormal movements or twitching**	During dosing reports	0 (0%)	0 (0%)	1 (3.6%)	NA – numbers too small
	At home reports	0 (0%)	1 (3.6%)	0 (0%)	
	Final visit reports	0 (0%)	2 (7.1%)	1 (3.6%)	
**Agitation**	During dosing reports	1 (3.2%)	0 (0%)	0 (0%)	
	At home reports	0 (0%)	1 (3.6%)	0 (0%)	
	Final visit reports	2 (6.5%)	2 (7.1%)	1 (3.6%)	
	**Overall participants reporting**	**3**	**3**	**1**	**X²=1.14, df=2, p=.57**
**Alertness**	During dosing reports	0 (0%)	2 (7.1%)	1 (3.6%)	
	At home reports	1 (3.2%)	2 (7.1%)	1 (3.6%)	

Side-effects – Table S6

**Reports of Side Effect**		**PF-04995274**, N = 31[1]	**Citalopram**, N = 28[1]	**Placebo**, N = 28[1]	**Group difference in number of participants who reported**
	Final visit reports	2 (6.5%)	1 (3.6%)	3 (11%)	
	**Overall participants reporting**	**3**	**5**	**4**	**X^2=0.5, df=2, p=.78**
**Dizziness**	During dosing reports	0 (0%)	3 (11%)	1 (3.6%)	
	At home reports	1 (3.2%)	3 (11%)	1 (3.6%)	
	Final visit reports	2 (6.5%)	4 (14%)	2 (7.1%)	
	**Overall participants reporting**	**3**	**8**	**3**	**X^2=3.57, df=2, p=.17**
**Hallucinations**	During dosing reports	0 (0%)	0 (0%)	0 (0%)	NA – numbers too small
	At home reports	0 (0%)	0 (0%)	0 (0%)	
	Final visit reports	0 (0%)	0 (0%)	0 (0%)	
**Impulse control problems**	During dosing reports	0 (0%)	1 (3.6%)	0 (0%)	NA – numbers too small
	At home reports	0 (0%)	0 (0%)	0 (0%)	
	Final visit reports	0 (0%)	0 (0%)	0 (0%)	
**Dry mouth**	During dosing reports	0 (0%)	1 (3.6%)	4 (14%)	
	At home reports	1 (3.2%)	6 (21%)	3 (11%)	
	Final visit reports	2 (6.5%)	6 (21%)	1 (3.6%)	
	**Overall participants reporting**	**2**	**7**	**6**	**X^2=2.8, df=2, p=.25**
**Headache**	During dosing reports	3 (9.7%)	2 (7.1%)	0 (0%)	
	At home reports	5 (16%)	2 (7.1%)	2 (7.1%)	
	Final visit reports	6 (19%)	3 (11%)	4 (14%)	
	**Overall participants reporting**	**9**	**5**	**4**	**X^2=2.33, df=2, p=.31**
**Back pain**	During dosing reports	0 (0%)	0 (0%)	0 (0%)	NA – numbers too small
	At home reports	0 (0%)	0 (0%)	2 (7.1%)	
	Final visit reports	0 (0%)	0 (0%)	1 (3.6%)	
**Other psychological side effects***	During dosing reports	1 (3.2%)	0 (0%)	0 (0%)	NA*
	At home reports	1 (3.2%)	3 (11%)	3 (11%)	
	Final visit reports	0 (0%)	0 (0%)	0 (0%)	
**Other physical side effects***	During dosing reports	0 (0%)	0 (0%)	0 (0%)	NA*
	At home reports	0 (0%)	2 (7.1%)	2 (7.1%)	
	Final visit reports	1 (3.2%)	3 (11%)	2 (7.1%)	

*Collated from optional free text, not systematically recorded, so statistical analyses have not been done.

Additional details on adverse events

Of the five participants who withdrew due to adverse events: one withdrew due to abdominal pain (placebo); one withdrew due to migraine (placebo); one withdrew due to stomach upset later deemed to be pre-menstrual symptoms (citalopram); one withdrew due to feeling nauseous, lightheaded and symptoms of derealisation (citalopram); and one withdrew due to disrupted sleep and dreaming, fever, headache and increased hopelessness (5-HT4 agonist). All symptoms resolved within 24 hours of discontinuation.

Including these five participants, in an Intention-To-Treat analysis, with a Last Observation Carried Forward approach used for missing data, led to results as follows: controlling for baseline severity, the citalopram group

scored significantly lower on the HAM-D at the final research visit compared to placebo ($F(1, 58)=6.032$, $p=0.02$, $\eta p2 = 0.09$), as did the PF-04995274 group ($F(1, 59)=7.41$, $p=0.009$, $\eta p2 = 0.11$).

Primary behavioural endpoints, reported in females participants only, split by hormonal contraception use – Table S7

	**Hormonal contraception use**	**No hormonal contraception**
PF-04995274	FERT Accuracy<br>Pos (72.8, 11.4) vs Neg (62.74, 8.52)<br><br>HAM-D<br>Screening (16.2, 2.52) vs End of Study (14.6, 5.21)<br><br>(n=5)	FERT Accuracy<br>Pos (73.8, 9.14) vs Neg (63.7, 11.4)<br><br>HAM-D<br>Screening (16.3, 2.48) vs End of Study (11, 2.66)<br><br>(n=13)
Citalopram	FERT Accuracy<br>Pos (74.4, 10.4) vs Neg (64.9, 9.74)<br><br>HAM-D<br>Screening (14.6, 1.04) vs End of Study (11.6, 3.12)<br><br>(n=6)	FERT Accuracy<br>Pos (72.6, 8.42) vs Neg (60.2, 13.5)<br><br>HAM-D<br>Screening (15.7, 3.11) vs End of Study (15.1, 5.67)<br><br>(n=12)
Placebo	FERT Accuracy<br>Pos (77.7, 8.29) vs Neg(64.8, 10.9)<br><br>HAM-D<br>Screening (17.5, 2.32) vs End of Study (17.7, 3.14)<br><br>(n=6)	FERT Accuracy<br>Pos (70.8, 13.3) vs Neg (64.5, 10.6)<br><br>HAM-D<br>Screening (16.9, 2.17) vs End of Study (16.6, 3.98)<br><br>(n=8)
